# Novel Nomograms to Predict Delayed Hyponatremia After Transsphenoidal Surgery for Pituitary Adenoma

**DOI:** 10.3389/fendo.2022.900121

**Published:** 2022-06-28

**Authors:** Kunzhe Lin, Ran Zeng, Shuwen Mu, Yinghong Lin, Shousen Wang

**Affiliations:** ^1^ Department of Neurosurgery, Affiliated Fuzhou First Hospital of Fujian Medical University, Fuzhou, China; ^2^ Fuzong Clinical Medical College of Fujian Medical University, Fuzhou, China; ^3^ Department of Neurosurgery, Shanghai Donglei Brain Hospital, Shanghai, China; ^4^ College of Integrated Chinese and Western Medicine, Fujian University of Traditional Chinese Medicine, Fuzhou, China; ^5^ Department of Neurosurgery, 900th Hospital, Fuzhou, China

**Keywords:** surgery, hyponatremia, magnetic resonance imaging, pituitary adenoma, nomogram

## Abstract

**Objective:**

This study aimed to develop a nomogram of clinical variables and magnetic resonance imaging scans to predict delayed hyponatremia after transsphenoidal surgery for pituitary adenoma.

**Methods:**

Patients who underwent transsphenoidal surgery for pituitary adenoma in Fuzong Clinical Medical College of Fujian Medical University between January 2012 and December 2020 were retrospectively investigated. Medical records, MRI findings, and laboratory examination results were recorded as candidate variable predictors of delayed hyponatremia. A nomogram to predict delayed hyponatremia was formulated based on the multivariable model of risk factors. The predictive accuracy and discriminative ability of the nomogram were assessed using the receiver operating characteristic (ROC) curve, calibration plot, and decision curve analyses. The model underwent prospective validation in three medical centers with patients who underwent transsphenoidal surgery for pituitary adenoma between January 2021 and February 2022.

**Results:**

The model that incorporated the postoperative length of “measurable pituitary stalk,” pituitary stalk deviation angle difference, postoperative diabetes insipidus, sinking depth of diaphragma sellae, and blood sodium level on the second postoperative day was developed and presented as the nomogram of the training cohort. The nomogram achieved area under the ROC curve (AUCs) of 0.806 and 0.849 for the training cohort and the testing cohort, respectively, and displayed good calibration. Decision curve analysis showed that the nomogram was clinically useful when the threshold probability was 13–96%.

**Conclusions:**

We developed a nomogram to evaluate the individualized prediction of delayed hyponatremia after transsphenoidal surgery for pituitary adenomas.

## Introduction

Pituitary adenoma is the most common sellar lesion, accounting for approximately 10–15% of intracranial tumors (1, 2). Most pituitary adenomas can be surgically removed using the transsphenoidal approach. Furthermore, for clinicians with rich surgical experience, the transsphenoidal approach can reduce surgical complications and shorten the length of hospital stay. Some research centers believe that patients without complications can be discharged from the hospital for home care 2–3 days post-operation (3).

Although transnasal sphenoid surgery (TSS) has become the preferred surgical method for the treatment of pituitary adenomas, the risk of postoperative delayed hyponatremia is still a concern. Delayed hyponatremia has been reported in literature, and its incidence fluctuates between 6.3% and 23.4% (4–8). The complication may be asymptomatic or may manifest as nausea, vomiting, changes in mental state, and seizures among others (5, 8). Delayed hyponatremia is a common cause of unplanned readmission within 30 days after TSS (9).Various research centers have attempted to predict the risk of postoperative delayed hyponatremia through the analysis of related factors; however, no consensus has been reached. Attempts to predict delayed hyponatremia after TSS by incorporating multiple variables have rarely been made.

A nomogram is a method of predicting the probability of a specific outcome by adding the scores of each risk factor. The result can be described as a continuous probability, and it can also graphically display the combined effect of each variable (10). Nomograms have been well applied in various fields (11). The use of nomograms can facilitate doctors in making clinical decisions, thereby improving patient prognosis. However, there have been no reports on the use of a nomogram to evaluate delayed hyponatremia after TSS. In our study, we developed a nomogram by combining clinical variables and magnetic resonance imaging (MRI) scans to predict delayed hyponatremia after TSS for pituitary adenoma.

## Materials and Methods

### Study Population

Retrospective analysis was conducted on the clinical data of patients with pituitary adenoma who underwent TSS in Fuzong Clinical Medical College of Fujian Medical University between January 2012 and December 2020. Nomograms were built using retrospective data as a training set. Prospective validation of the nomograms was then performed in three medical centers (Fuzong Clinical Medical College of Fujian Medical University, Affiliated Fuzhou First Hospital of Fujian Medical University and Shanghai Donglei Brain Hospital) with patients who underwent transsphenoidal surgery for pituitary adenoma between January 2021 and February 2022. The study design was approved by the ethics committee of all the medical centers. All study participants provided informed consent upon admission. The inclusion criteria were as follows: 1) patients undergoing first-time pituitary surgery; 2) patients with pituitary adenoma undergoing TSS under a microscope or endoscope; and 3) Presence of an MRI review 3 days post-operation with complete clinical data. The exclusion criteria were: 1) history of pituitary surgery or radiotherapy; and 2) pituitary adenoma combined with other sellar lesions, such as Rathke’s cleft cyst.

### Perioperative Management

The management of patients with pituitary adenoma is multidisciplinary (neurosurgery, endocrinology, radiology, ophthalmology, and anesthesiology). We adopted a unified and standardized management method. Physiological treatment was required for the presence of secondary hypothyroidism and secondary adrenal cortex hypofunction. We routinely recorded the 24-h postoperative input and output of the patients and monitored electrolyte concentrations and the specific gravity of the urine. Blood sodium tests were performed at 8:00 am on the day before surgery and daily after surgery until discharge. Patients with pituitary adenomas were supplemented with a stress dose of glucocorticoids (except for those with Cushing’s disease) on the day of surgery, and the dose of glucocorticoids was gradually tapered to a physiological replacement dose postoperatively. Patients were supplemented with physiological requirements after the surgery, and intravenous fluids were reduced after the patients resumed their diet. Patients with diabetes insipidus (DI) were provided with free drinking water, and desmopressin was administered when necessary.

The diagnostic criteria for DI included (12): (1) polyuria (urinary output > 300 ml/h for 3 h); (2) urine specific gravity < 1.005; (3) at least one relative criterion of either: excessive thirst, serum osmolality > 300 mosmol/kg or serum sodium > 145 mmol/L.

Hyponatremia was defined as a serum sodium concentration <135 mmol/L (13). Delayed hyponatremia was defined as hyponatremia that occurred on or after the third day after TSS (9, 14).

### Study Predictors

**Figure 1:**
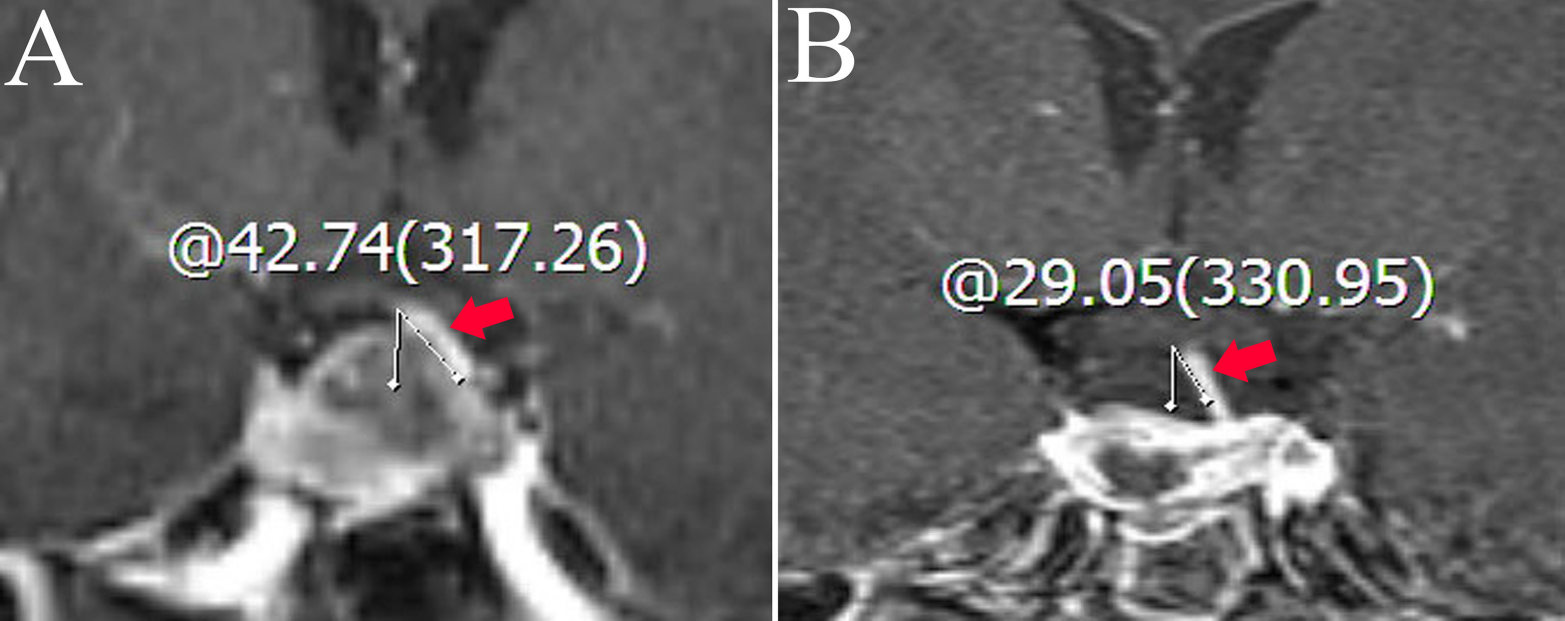
Coronal contrast-enhanced image shows the measurement of the deviation angle of the pituitary stalk. The red arrow points to the pituitary stalk. **(A)** The patient’s pituitary stalk is 42.74° to the left preoperatively. **(B)** The patient’s pituitary stalk is 29.05° to the left postoperatively.

**Figure 2:**
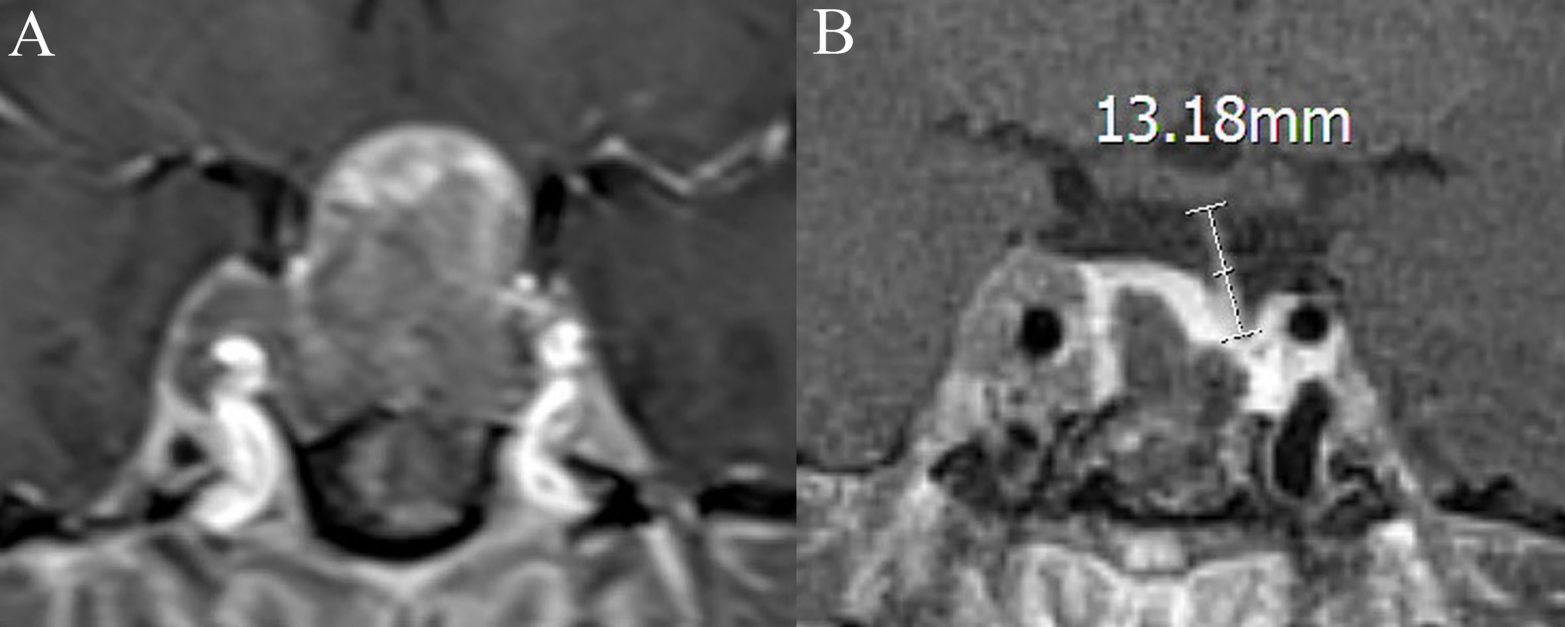
Coronal contrast-enhanced image shows the measurement of the length of the “measurable pituitary stalk.” **(A)** The pituitary stalk is compressed preoperative, and its length is difficult to measure. The length of the “measurable pituitary stalk” preoperatively is 0. **(B)** The length of the “measurable pituitary stalk” postoperatively is 13.18 mm.

**Figure 3:**
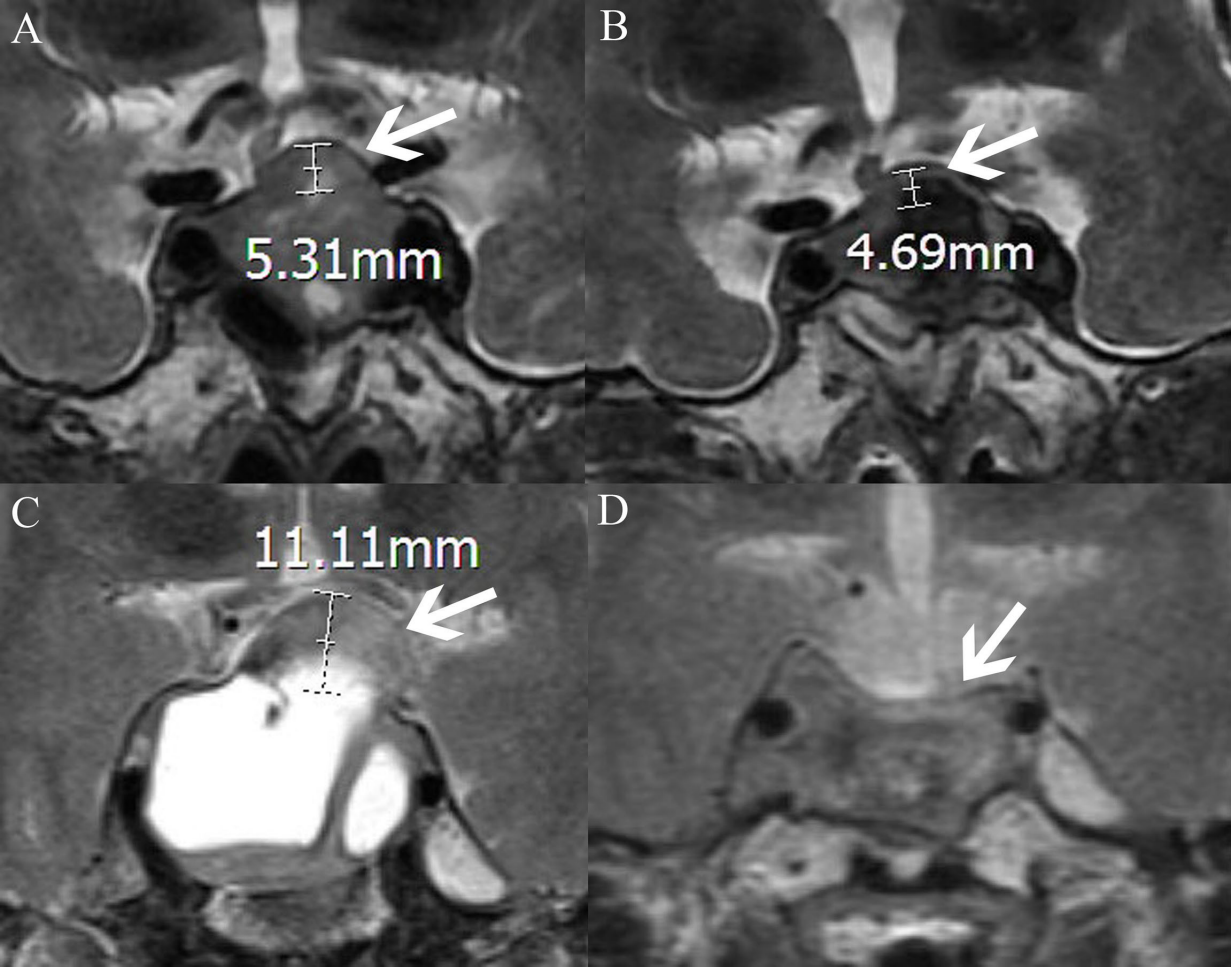
MRI-T2WI shows the measurement of the elevation of the diaphragm sellae (the distance between the plane where the diaphragm sellae starts to rise and the plane of the highest point of the diaphragm sellae; the white arrow points to the diaphragm sellae, AB and CD, respectively, represent the same patient). **(A)** The elevation of the diaphragm sellae before surgery is 5.31 mm. **(B)** The elevation of the diaphragm sellae after surgery is 4.69 mm. The diaphragma sellae sinking depth is the difference between the two values (5.31–4.69 mm). **(C)** The elevation of the diaphragm sellae before surgery is 11.11 mm. **(D)** The elevation of the diaphragm sellae after surgery is 0 mm. The diaphragma sellae sinking depth is the difference between the two values (11.11–0 mm).

Medical records, MRI findings, and laboratory examination results were recorded as candidate variable predictors. Relevant variables obtained from the medical records included age, sex, cerebrospinal fluid leakage (intraoperative), and DI (postoperative). Laboratory examination-related variables included pathological type, endocrine levels, and electrolyte levels. MRI-related variables included diaphragma sellae sinking depth; tumor height, volume, and invasiveness; intratumoral cysts or hematoma; location of the posterior pituitary bright spot (PPBS); extent of tumor resection; pituitary stalk deviation angle; and length of the “measurable pituitary stalk.” The angle at which the starting part of the pituitary stalk deviates from the midline was defined as the deviation angle of the pituitary stalk (**Figure 1**) (15). The difference between the deviation angle of the pituitary stalk preoperatively and postoperatively was calculated (i.e., pituitary stalk deviation angle difference). The length of the “measurable pituitary stalk” and the sinking depth of the diaphragma sellae were measured and calculated according to Lin’s method (**Figures 2**, **3**) (16). Tumor volume was calculated using the platform-like volume calculation formula (17). Tumor resection was classified based on the extent of resection as total, subtotal, and partial (18). Approximately 3–6 months postoperatively, MRI images were reviewed to confirm the degree of tumor resection. Patients with Knosp Grades 3 or 4 were defined as having cavernous sinus invasion (19). A neurosurgeon and a neuroradiologist (with more than 10 years of clinical experience) independently measured the relevant indicators of MRI (before surgery and on the third postoperative day) and took the average value for statistical analysis. Disagreements regarding imaging findings were resolved through discussion until consensus was achieved.

### Statistical Analyses

Statistical analyses were performed using the R software (Version 4.1.0; https://www.R-project.org). The least absolute shrinkage and selection operator (LASSO) logistic regression was applied to select predictors and develop the nomogram. Features with nonzero coefficients in the LASSO regression model were selected. Multivariable logistic regression analysis was used to build a prediction model by incorporating the feature selected in the LASSO regression model. The discrimination of the nomogram was assessed using the receiver operating characteristic (ROC) curve. Furthermore, the calibration curve was used to evaluate the accuracy of the prediction. The clinical practicability was evaluated by decision curve analysis (DCA).

## Results

### Patients’ Characteristics

Overall, 400 patients with pituitary adenomas were included in the training cohort (**Table 1**). The age of the patients ranged from 18 to 83 years, with a mean age of 48.94 ± 13.31 years. Of the patients, 78 developed postoperative delayed hyponatremia while 322 patients did not. The clinical, laboratory, and radiological features of delayed hyponatremia and normonatremia groups are shown in **Supplementary Table 1**. Out of the 78 patients with delayed hyponatremia, 49 presented clinical symptoms of hyponatremia, including seizures, vomiting, headache, poor appetite, and fatigue.

**Table 1:** Comparison of the characteristics between the training and validation cohorts.

Factors	Training cohort (n = 400)	Validation cohort (n = 107)	*P* value
**Baseline information**			
Age, yrs.	48.94 ± 13.31	49.61 ± 13.47	0.645
Sex			0.101
Male	210 (52.5)	46 (43.0)	
Female	190 (47.5)	61 (57.0)	
**Medical records**			
Intraoperative cerebrospinal fluid leaks			0.171
Yes	332 (83.0)	82 (76.6)	
No	68 (17.0)	25 (23.4)	
Postoperative DI			0.155
Yes	206 (51.5)	64 (59.8)	
No	194 (48.5)	43 (40.2)	
**Radiological features**			
Tumor volume, cm^3^	5.28 ± 6.27	5.25 ± 6.21	0.524
Tumor height, mm	23.12 (9.50)	22.19 (9.31)	0.364
Intratumoral cysts or hematoma			0.735
Yes	144 (36.0)	36 (33.6)	
No	256 (64.0)	71 (66.4)	
Location of the PPBS			0.263
Superior parts	176 (44.0)	47 (43.9)	
Inferior parts	171 (42.8)	43 (40.2)	
Superior and inferior parts	18 (4.5)	2 (1.9)	
None	35 (8.8)	15 (14.0)	
Invasiveness			0.114
Yes	348 (87.0)	86 (80.4)	
No	52 (13.0)	21 (19.6)	
Extent of tumor resection			0.676
total resection	339 (84.8)	87 (81.3)	
subtotal resection	20 (5.0)	7 (6.5)	
partial resection	41 (10.2)	13 (12.1)	
Diaphragma sellae sinking depth, mm	3.75 (4.29)	2.93 (4.05)	0.079
Pituitary stalk deviation angle difference	11.16 (14.87)	9.73 (13.78)	0.368

Data are presented as number of patients (%). PPBS, posterior pituitary bright spot; DI, diabetes insipidus.

### Predictors’ Selection

**Figure 4:**
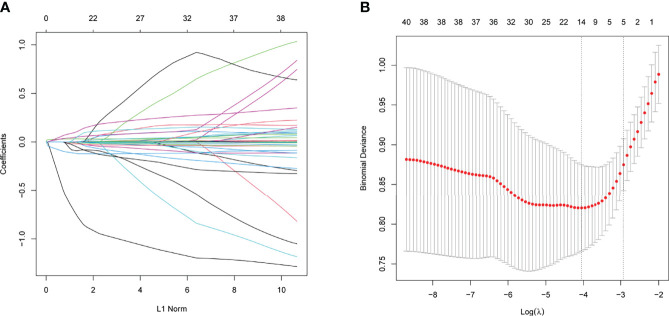
The variables selection using the LASSO logistic regression model. **(A)** Lasso coefficient profiles of the features. A coefficient profile plot was produced against the log (lambda) sequence. **(B)** A vertical line was drawn at the value selected using tenfold cross-validation, where optimal values by using the minimum criteria and the 1 standard error of the minimum criteria.

Regarding demographic, MRI, and laboratory factors, 32 features (**Supplementary Table 2**) were reduced to five potential predictors based on 400 patients in the training cohort and were with nonzero coefficients in the LASSO regression model (**Figure 4**). These factors included postoperative length of the “measurable pituitary stalk”, pituitary stalk deviation angle difference, postoperative DI, sinking depth of diaphragma sellae, and blood sodium level on the second day postoperatively.

### Development of Prediction Model in the Training Cohort

**Figure 5:**
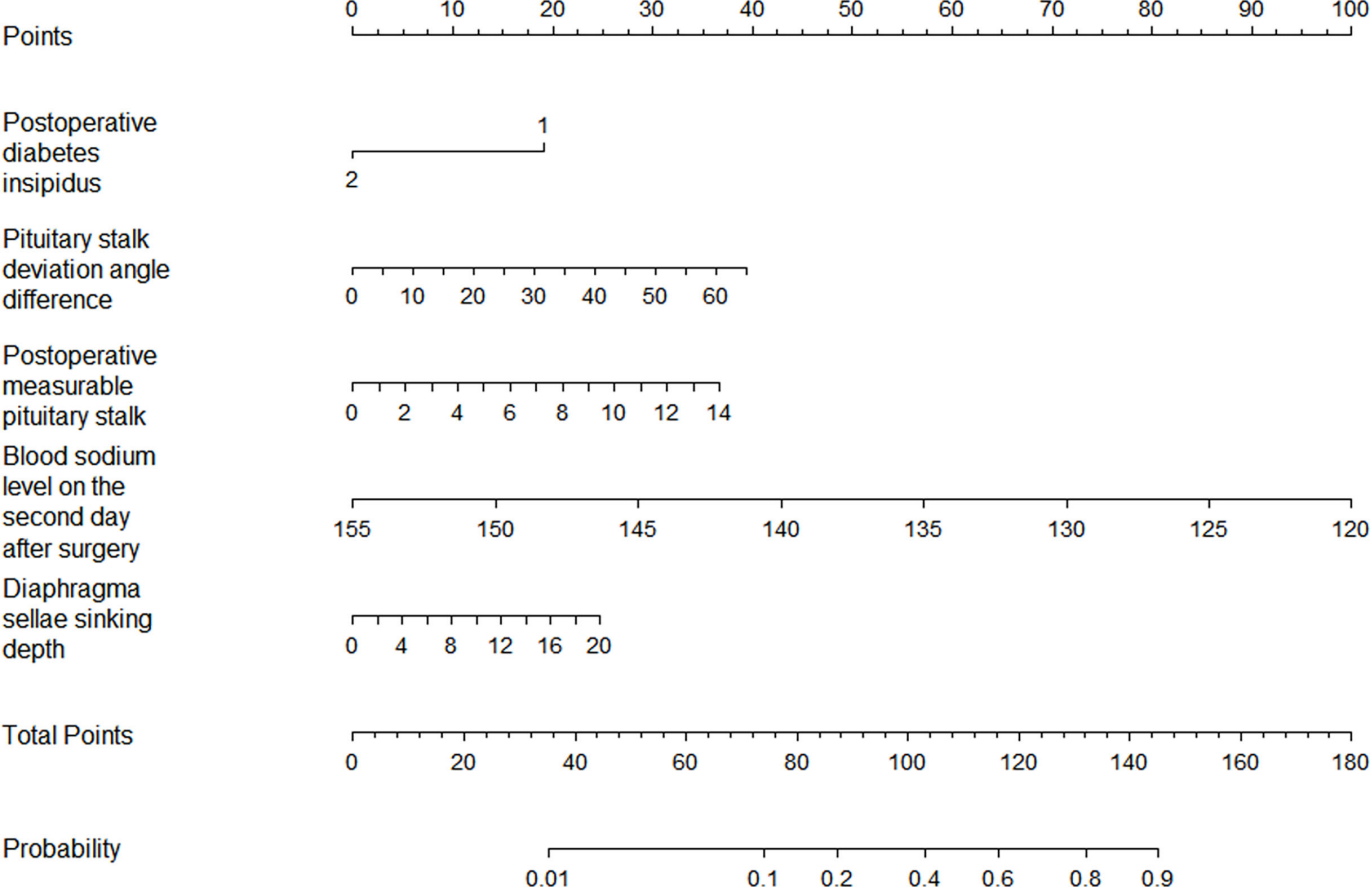
The nomogram for the prediction of delayed hyponatremia after TSS. The predictor points can be found on the uppermost point scale that correspond to each patient variable and can be added up. The total points projected to the bottom scale indicate the risk of delayed hyponatremia. (For the diabetes insipidus variable, 1 means “No,” 2 means “Yes”).

The results of the logistic regression analysis of postoperative length of the “measurable pituitary stalk”, pituitary stalk deviation angle difference, postoperative DI, sinking depth of diaphragma sellae, and blood sodium level on the second postoperative day are shown in **Table 2**. The model that incorporated the aforementioned independent predictors was developed and presented as the nomogram of the training cohort (**Figure 5**).

**Table 2:** Logistic regression analysis of risk of postoperative delayed hyponatremia onset.

Factors	Odds ratio	95% C I	*P* value
Diaphragma sellae sinking depth	1.079	1.001, 1.163	0.045
Postoperative DI	0.305	0.162, 0.555	< 0.001
Blood sodium level on the second day after surgery	0.838	0.771, 0.905	< 0.001
The deviation angle difference of the pituitary stalk	1.038	1.017, 1.060	< 0.001
Postoperative length of the “measurable pituitary stalk”	1.175	1.057, 1.315	0.003

DI, diabetes insipidus.

### Validation of the Nomogram

**Figure 6:**
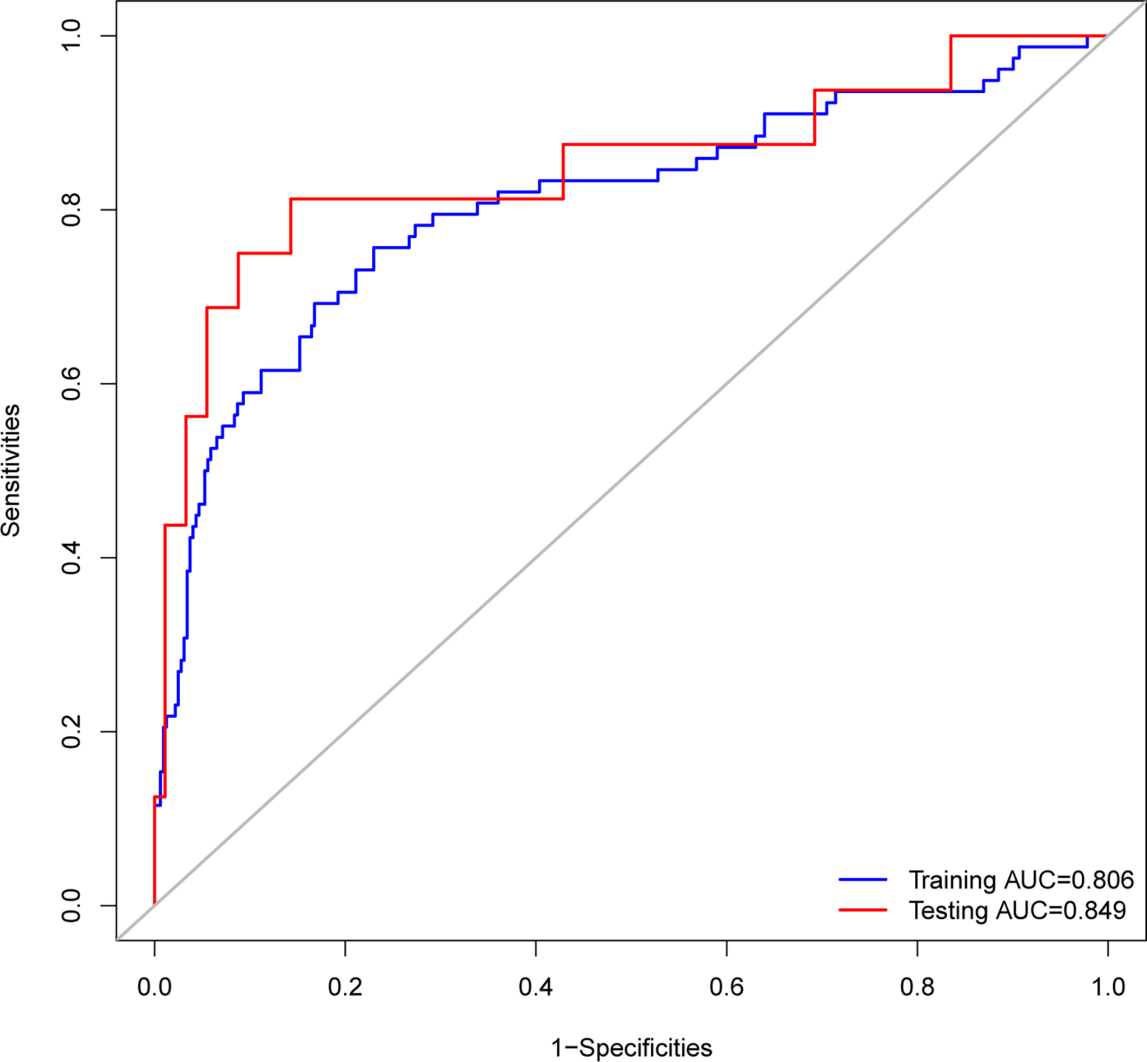
Receiver operating characteristic (ROC) curves of the nomograms. The nomogram had good discriminative power with area under ROC curve of 0.806 and 0.849 for the training cohort and the testing cohort, respectively.

**Figure 7:**
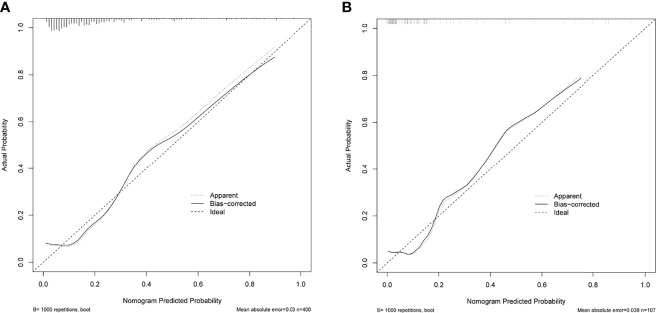
Calibration curves of the nomogram for predicting delayed hyponatremia after TSS. The x-axis represents the predicted delayed hyponatremia risk. The y-axis represents the actual delayed hyponatremia risk. The diagonal dotted line represents a perfect prediction by an ideal model. The solid green line represents the performance of the nomogram, of which a closer fit to the diagonal dotted line represents a better prediction. **(A)** Calibration curve of the nomogram in the training cohort. **(B)** Calibration curve of the nomogram in the testing cohort.

ROC analysis showed that the area under the ROC curve (AUC) of the nomogram model in the training cohort was 0.806 (**Figure 6**). The calibration curve revealed that the bias-corrected line, which presented the performance of the bootstrap-corrected nomogram, was close to ideal, representing good predictions (**Figure 7**). The p-value of the Hosmer–Lemeshow test was 0.356, which indicated a good calibration power of the model.

**Figure 8:**
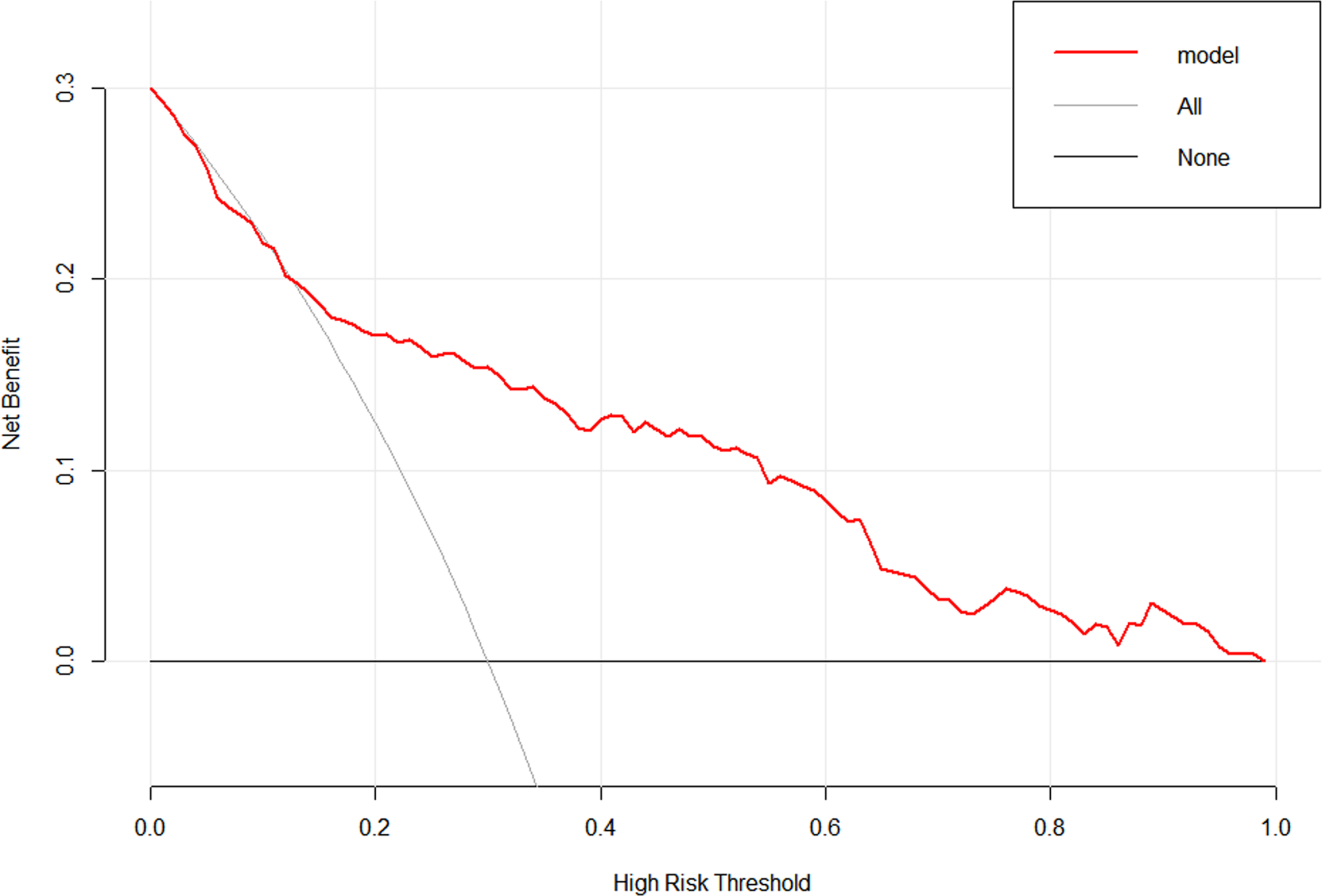
Decision curve analysis for the nomogram. The y-axis measures the net benefit. The red line represents the nomogram. The thin solid line represents the assumption that all patients have delayed hyponatremia. The thick solid line represents the assumption that no patients have delayed hyponatremia.

To evaluate the clinical practicability of the model, DCA was performed (**Figure 8**), which means that if the threshold probability was 13–96%, using the nomogram in the current study to predict delayed hyponatremia added more benefit than the treat-all-patients scheme or the treat-none scheme.

### Prospective Validation of the Nomogram

In total, 107 patients with pituitary adenomas from the three medical centers were included in the testing cohort. The clinical, laboratory, and radiological features of delayed hyponatremia and normonatremia groups are shown in **Supplementary Table 3**. **Table 1** shows comparison of the characteristics between the training and validation cohorts, and no significant differences were observed in the baseline characteristics of the two cohorts. Good calibration was also observed for the probability of delayed hyponatremia in the testing cohort, depicted in **Figure 7**, with the nomogram achieving AUC of 0.849.

## Discussion

We developed and validated a novel prediction tool for delayed hyponatremia after TSS for pituitary adenoma. Incorporating laboratory and MRI factors into an easy-to-use nomogram facilitates individualized prediction of delayed hyponatremia after TSS. The nomogram achieved an AUC of 0.849 for the testing cohort and was well calibrated. Neurosurgeons can easily utilize this nomogram in clinical practice to provide more accurate predictions of delayed hyponatremia after surgery in patients with pituitary adenomas.

The pathophysiology of delayed hyponatremia after TSS remains unknown. Studies have shown that the main cause of delayed hyponatremia is mechanical iatrogenic damage to the hypothalamus-neurohypophysis system, resulting in the uncontrolled release of the antidiuretic hormone (ADH) (20, 21). Previously, we proposed that descent of the diaphragma sellae could be a possible cause of hypothalamus-neurohypophysis tract injury (22). After tumor removal, the subsidence of the diaphragma sellae usually pulls the pituitary stalk, which can lead to abnormal secretion of ADH and cause delayed hyponatremia (16). Previous studies used statistical methods, such as univariate and multivariate analysis to determine the predictors. In this study, MRI-related predictive indicators were screened through LASSO regression. The final included indicators were diaphragma sellae sinking depth, postoperative measurable pituitary stalk, and pituitary stalk deviation angle difference. Furthermore, it can also be suggested that early postoperative MRI review helps predict the occurrence of delayed hyponatremia. According to the nomogram model, multiple predictors can be integrated to determine the probability of a patient developing delayed hyponatremia.

The position and length of the pituitary stalk may change along with the surrounding structure (23). Due to the slow growth of pituitary adenoma, the position and shape of the pituitary stalk changes as it is pushed and compressed. During TSS, with the removal of the tumor, the position and shape of the pituitary stalk may vary again or it may be pulled and damaged. Differences in the pituitary stalk deviation angle and postoperative measurements of the pituitary stalk are indirect evidence of changes in the position or shape of the pituitary stalk. The larger the difference between the deviation angles of the pituitary stalk, the more obvious the change in the position of the pituitary stalk. Owing to the influence of the tumor, it is difficult to accurately measure the entire pituitary stalk preoperatively. The measurable pituitary stalk pre- and postoperatively were taken into account and the results showed no difference in the preoperative measurable pituitary stalk length between the delayed hyponatremia group and the normonatremia group, while the postoperative measurable pituitary stalk length was longer in the hyponatremia group, indicating that it could be used to predict the occurrence of delayed postoperative hyponatremia. This was incorporated into the nomogram model. This suggests that the longer the postoperative “measurable pituitary stalk,” the more obvious the change in the position or morphology of the pituitary stalk, and the damage to the pituitary stalk may be greater, resulting in abnormal secretion of ADH, and subsequently delayed hyponatremia.

Multivariate analysis and nomogram model also showed that the emergence of postoperative DI indicated a lower risk of delayed hyponatremia. The study by Patel also confirms this view (5). Postoperative DI incidence after TSS ranges from 12.5% to 45.8% (24, 25). DI is mostly transient in patients with pituitary adenoma who were operated *via* the transsphenoidal approach, it occurs within 24–48 h postoperatively, and resolves within a couple of days (26). Additionally, DI is caused by the mechanical irritation of the posterior pituitary gland, pituitary stalk, hypothalamus, and infundibulum (or by shock of the neurohypophyseal axons) by surgery, resulting in decreased ADH secretion (24, 27, 28). Postoperative DI and delayed hyponatremia can develop isolated or occur successively in a patient. They represent the range of manifestation of one disease (disturbances in ADH secretion following manipulation of the neurohypophysis) (29). According to the degree of damage, postoperative patients may exhibit fluctuations in ADH secretion termed “biphasic” and “triphasic” response, and syndrome of inappropriate antidiuretic hormone secretion (SIADH) likely represents an “isolated second phase” of the triphasic response (3, 27, 30). In this study, there were 25 and 65 patients who had combined DI-delayed hyponatremia and isolated delayed hyponatremia in the training cohort, respectively. Therefore, for those who did not develop DI postoperatively, we must analyze whether delayed hyponatremia will further occur.

In previous studies, the reported predictors of delayed hyponatremia were age (7, 8, 31), sex (14), preoperative hypopituitarism (32), and intraoperative CSF leak (14). These predictors were not included in the nomogram model, and it was difficult to assess the degree of damage to the neurohypophysis. Based on the MRI findings, we verified that the changes in the position of the diaphragm sellae and pituitary stalk before and after surgery could predict the occurrence of delayed hyponatremia after surgery. In addition, patients with low blood sodium levels on the second postoperative day were likely to have a higher risk of delayed hyponatremia. This observation is consistent with the results of Tomita (8). Yoon (7) showed that patients with low blood sodium levels on the first and second days after surgery had a higher risk of delayed hyponatremia. The lower serum sodium concentration in the early postoperative period may be the result of excessive ADH secretion (8). Therefore, restricting fluid intake may be expected to reduce the occurrence of delayed hyponatremia. Burke (21) confirmed that fluid restriction impacts the stabilization of postoperative serum sodium concentrations, limiting readmissions for hyponatremia.

After the model was established in this study, it was verified through three levels of ROC curve, calibration curve, and DCA. This nomogram had excellent discriminative power with AUCs of 0.806 and 0.849 for the training cohort and the testing cohort, respectively, and had an obvious net benefit for almost all threshold probabilities as the decision curve indicated. The calibration curve showed no significant difference between the predicted and the observed probability. To the best of our knowledge, this is the first study to establish a nomogram that could predict the risk of delayed hyponatremia after TSS. With the help of the nomogram, neurosurgeons could accurately identify potential patients who would develop delayed hyponatremia after surgery and intervene in time according to risk factors.

There are several limitations in our study. First, potential bias was difficult to avoid in a retrospective study. Second, details of hormone testing, including testing reagents and testing methods, were not standardized in multicenter studies. Third, water restriction may help to reduce the occurrence of delayed hyponatremia, which was not investigated in this study. Fourth, early postoperative MRI examination is beneficial, but was not a standardized procedure and was difficult to popularize in some medical centers.

## Conclusions

The nomogram constructed in this study will prove to be a useful and convenient tool for the evaluation of the risk of delayed hyponatremia after TSS for pituitary adenomas and could provide individualized prediction. Through individual risk assessment, clinicians can adopt necessary medical interventions in a targeted manner.

## Data Availability Statement

The original contributions presented in the study are included in the article/**Supplementary Material**. Further inquiries can be directed to the corresponding author.

## Ethics Statement

The studies involving human participants were reviewed and approved by Fuzong Clinical Medical College of Fujian Medical University, Affiliated Fuzhou First Hospital of Fujian Medical University and Shanghai Donglei Brain Hospital. The patients/participants provided their written informed consent to participate in this study.

## Author Contributions

KL: Data curation, Writing original draft. RZ: Data curation, Writing original draft. SM: Data retrieval, statistics. YL, Data retrieval, statistics. SW: designed the study and revised the manuscript. All authors read and approved the final manuscript.

## Funding

This work was supported by the Natural Science Foundation of Fujian Province [2021J011306] and Department of Science and Technology of Fuzhou City [2021-S-180].

## Conflict of Interest

The authors declare that the research was conducted in the absence of any commercial or financial relationships that could be construed as a potential conflict of interest.

## Publisher’s Note

All claims expressed in this article are solely those of the authors and do not necessarily represent those of their affiliated organizations, or those of the publisher, the editors and the reviewers. Any product that may be evaluated in this article, or claim that may be made by its manufacturer, is not guaranteed or endorsed by the publisher.
