# Supplementary material for: Novel Nomograms to Predict Delayed Hyponatremia After Transsphenoidal Surgery for Pituitary Adenoma
Source: Front Endocrinol (Lausanne). 2022 Jun 28;13:900121. doi: 10.3389/fendo.2022.900121 (PMC9273860; doi:10.3389/fendo.2022.900121)
Supplement: Supplementary file 1 [file DataSheet_1.doc]

| Supplementary Table 1 The clinical, laboratory, and radiological features of delayed hyponatremia and normonatremia groups in the training cohorts | | | |
| --- | --- | --- | --- |
| Factors | Delayed hyponatremia  (n=78) | Normonatremia  (n=322) | *P* value |
| **Baseline information** |  |  |  |
| Age, yrs | 50.96±13.84 | 48.45±13.16 | 0.135 |
| Sex |  |  | 0.695 |
| Male | 43 (55.1) | 167 (51.9) |  |
| Female | 35(44.9) | 155(48.1) |  |
| **Medical records** |  |  |  |
| Intraoperative cerebrospinal fluid leaks |  |  | 0.677 |
| Yes | 15(19.2) | 53(16.5) |  |
| No | 63(80.8) | 269(83.5) |  |
| Postoperative DI |  |  | 0.002 |
| Yes | 25(32.1) | 169(52.5) |  |
| No | 53(67.9) | 153(47.5) |  |
| **Radiological features** |  |  |  |
| Tumor volume, cm^3^ | 5.80±5.03 | 5.65+6.54 | 0.849 |
| Tumor height, mm | 23.71±9.65 | 22.98±9.47 | 0.541 |
| Intratumoral cysts or hematoma |  |  | 0.369 |
| Yes | 32(41.0) | 112(34.8) |  |
| No | 46(59.0) | 210(65.2) |  |
| Location of the PPBS |  |  | 0.350 |
| Superior parts | 37(47.4) | 139(43.2) |  |
| Inferior parts | 29(37.2) | 142(44.1) |  |
| Superior and inferior parts | 6(7.7) | 12(3.7) |  |
| None | 6(7.7) | 29(9) |  |
| Invasiveness |  |  | 0.866 |
| Yes | 9(11.5) | 42(13.0) |  |
| No | 69(88.5) | 280(87.0) |  |
| Extent of tumor resection |  |  | 0.103 |
| total resection | 70(89.7) | 269(83.5) |  |
| subtotal resection | 5(6.4) | 15(4.7) |  |
| partial resection | 3(3.8) | 38(11.8) |  |
| Diaphragma sellae sinking depth, mm | 6.04±5.23 | 3.19±3.84 | < 0.001 |
| Pituitary stalk deviation angle difference, (°) | 21.44±17.96 | 8.68±12.87 | < 0.001 |
| Length of the “measurable pituitary stalk”, mm  （Preoperative） | 3.02±3.32 | 3.91±3.54 | 0.043 |
| Length of the “measurable pituitary stalk”, mm  （Postoperative） | 7.60±3.10 | 6.03±2.95 | < 0.001 |
| Preoperative shape of pituitary stalk |  |  | 0.047 |
| curved | 46(59.0) | 147(45.7) |  |
| straight | 32(41.0) | 175(54.3) |  |
| Postoperative shape of pituitary stalk |  |  | 0.252 |
| curved | 9(11.5) | 57(17.7) |  |
| straight | 69(88.5) | 265(82.3) |  |
| **laboratory examination variables** |  |  |  |
| Pathological tumor type |  |  | 0.280 |
| Gonadotropinoma | 32(41.0) | 90(28.0) |  |
| Null-cell adenoma | 17(21.8) | 82(25.5) |  |
| PRL cell adenoma | 12(15.4) | 56(17.4) |  |
| GH cell adenoma | 5(6.4) | 26(8.1) |  |
| ACTH cell adenoma | 3(3.8) | 33(10.2) |  |
| Plurihormona | 8(10.3) | 33(10.2) |  |
| TSH cell adenoma | 1(1.3) | 2(0.6) |  |
| Preoperative FT3, pmol/L | 3.94±0.90 | 4.44±1.74 | 0.014 |
| PostoperativeFT3, pmol/L | 3.11±0.69 | 3.33±1.16 | 0.102 |
| Preoperative FT4, pmol/L | 12.49±3.36 | 13.60±3.81 | 0.019 |
| Postoperative FT4, pmol/L | 13.23±3.46 | 14.58±4.92 | 0.023 |
| Preoperative TSH, mIU/L | 1.84±1.99 | 1.66±1.30 | 0.333 |
| Postoperative TSH, mIU/L | 0.75±0.69 | 0.81±0.84 | 0.583 |
| Preoperative cortisol, nmol/L | 12.58±10.45 | 13.91±10.46 | 0.313 |
| Postoperative cortisol, nmol/L | 24.22±14.00 | 25.88±16.37 | 0.41 |
| Preoperative ACTH, pg/ml | 24.28±18.10 | 33.10±37.24 | 0.043 |
| Postoperative ACTH, pg/ml | 27.77±25.93 | 29.20±32.22 | 0.715 |
| Preoperative serum sodium level, mEq/L | 139.48±5.02 | 140.40±3.10 | 0.043 |
| Serum sodium on the first day after surgery, mEq/L | 138.79±3.83 | 140.17±3.17 | 0.001 |
| Serum sodium on the second day after surgery, mEq/L | 138.37±4.99 | 140.78±3.29 | < 0.001 |
| The difference in serum sodium levels before and 1st day after surgery, mEq/L | 0.69±4.81 | 0.23±3.62 | 0.345 |
| The difference inserum sodium levels before and 2nd day after surgery, mEq/L | 1.11±6.01 | -0.38±3.71 | 0.001 |
| Data are presented as number of patients (%). PRL, Prolactin; GH, growth hormone; ACTH, adrenocorticotropic hormone; TSH, thyroid-stimulating hormone; FT3, Free triiodothyronine; FT4, free thyroxine; PPBS, posterior pituitary bright spot; DI, diabetes insipidus. | | | |

| Supplementary Table 2 32 features based on 400 patients in the training cohort  included in the LASSO regression model | | | | |  |
| --- | --- | --- | --- | --- | --- |
| Factors  (n=32) | | the training cohort  (n=400) | | |  |
| Age, yrs | | 48.93±13.31 | | |  |
| Sex | |  | | |  |
| Male | | 210 (52.5) | | |  |
| Female | | 190(47.5) | | |  |
| Intraoperative cerebrospinal fluid leaks | |  | | |  |
| Yes | | 68(17.0) | | |  |
| No | | 322(83.0) | | |  |
| Postoperative DI | |  | | |  |
| Yes | | 194(48.5) | | |  |
| No | | 206(51.5) | | |  |
| Tumor volume, cm^3^ | | 5.56±6.27 | | |  |
| Tumor height, mm | | 23.12±9.49 | | |  |
| Intratumoral cysts or hematoma | |  | | |  |
| Yes | | 144(36.0) | | |  |
| No | | 256(64.0) | | |  |
| Location of the PPBS | |  | | |  |
| Superior parts | | 176(44.0) | | |  |
| Inferior parts | | 171(42.7) | | |  |
| Superior and inferior parts | | 18(4.5) | | |  |
| None | | 35(8.8) | | |  |
| Invasiveness | |  | | |  |
| Yes | | 51(12.7) | | |  |
| No | | 349(87.3) | | |  |
| Extent of tumor resection | |  | | |  |
| total resection | | 339(84.7) | | |  |
| subtotal resection | | 20(5.0) | | |  |
| partial resection | | 41(10.3) | | |  |
| Diaphragma sellae sinking depth, mm | | 3.74±4.28 | | |  |
| Pituitary stalk deviation angle difference, (°) | | 11.16±14.86 | | |  |
| Length of the “measurable pituitary stalk”, mm  （Preoperative） | | 3.73±3.51 | | |  |
| Length of the “measurable pituitary stalk”, mm  （Postoperative） | | 6.33±3.04 | | |  |
| Preoperative shape of pituitary stalk | |  | | |  |
| curved | | 193(48.3) | | |  |
| straight | | 207(51.7) | | |  |
| Postoperative shape of pituitary stalk | |  | | |  |
| curved | | 66(16.5) | | |  |
| straight | | 334(83.5) | | |  |
| **laboratory examination variables** | |  | | |  |
| Pathological tumor type | |  | | |  |
| Gonadotropinoma | | 122(30.5) | | |  |
| Null-cell adenoma | | 99(24.7) | | |  |
| PRL cell adenoma | | 68(17.0) | | |  |
| GH cell adenoma | | 31(7.8) | | |  |
| ACTH cell adenoma | | 36(9.0) | | |  |
| Plurihormona | | 41(10.2) | | |  |
| TSH cell adenoma | | 3(0.8) | | |  |
| Preoperative FT3, pmol/L | | 4.34±1.62 | | |  |
| PostoperativeFT3, pmol/L | | 3.29±1.08 | | |  |
| Preoperative FT4, pmol/L | | 13.38±3.75 | | |  |
| Postoperative FT4, pmol/L | | 14.31±4.70 | | |  |
| Preoperative TSH, mIU/L | | 1.59±1.45 | | |  |
| Postoperative TSH, mIU/L | | 0.79±0.81 | | |  |
| Preoperative cortisol, nmol/L | | 13.64±10.45 | | |  |
| Postoperative cortisol, nmol/L | | 25.55±15.93 | | |  |
| Preoperative ACTH, pg/ml | | 31.38±34.51 | | |  |
| Postoperative ACTH, pg/ml | | 28.92±31.06 | | |  |
| Preoperative serum sodium level, mEq/L | | 140.21±3.57 | | |  |
| Serum sodium on the first day after surgery, mEq/L | | 139.89±3.35 | | |  |
| Serum sodium on the second day after surgery, mEq/L | | 140.31±3.79 | | |  |
| The difference in serum sodium levels before and 1st day after surgery, mEq/L | | 0.32±3.88 | | |  |
| The difference inserum sodium levels before and 2nd day after surgery, mEq/L | | 0.09±4.29 | | |  |
| Data are presented as number of patients (%). PRL, Prolactin; GH, growth hormone; ACTH, adrenocorticotropic hormone; TSH, thyroid-stimulating hormone; FT3, Free triiodothyronine; FT4, free thyroxine; PPBS, posterior pituitary bright spot; DI, diabetes insipidus. | | | | |  |
| Supplementary Table 3 The clinical, laboratory, and radiological features of delayed hyponatremia and normonatremia groups in the validation cohorts | | | | | |
| Factors | Delayed hyponatremia  (n=16) | | Normonatremia  (n=91) | *P* value | |
| **Baseline information** |  | |  |  | |
| Age, yrs | 52.00±12.64 | | 49.19±13.63 | 0.444 | |
| Sex |  | |  | 0.734 | |
| Male | 8 (50.0) | | 38 (41.8) |  | |
| Female | 8 (50.0) | | 53 (58.2) |  | |
| **Medical records** |  | |  |  | |
| Intraoperative cerebrospinal fluid leaks |  | |  | 0.879 | |
| Yes | 3 (18.8) | | 22 (24.2) |  | |
| No | 13 (81.2) | | 69 (75.8) |  | |
| Postoperative DI |  | |  | 0.969 | |
| Yes | 7 (43.8) | | 36 (39.6) |  | |
| No | 9 (56.2) | | 55 (60.4) |  | |
| **Radiological features** |  | |  |  | |
| Tumor volume, cm^3^ | 5.33±5.18 | | 5.23±6.40 | 0.952 | |
| Tumor height, mm | 22.30 ±10.49 | | 22.17±9.15 | 0.958 | |
| Intratumoral cysts or hematoma |  | |  | 0.018 | |
| Yes | 10 (62.5) | | 26 (28.6) |  | |
| No | 6 (37.5) | | 65 (71.4) |  | |
| Location of the PPBS |  | |  | 0.902 | |
| Superior parts | 8 (50.0) | | 39 (42.9) |  | |
| Inferior parts | 6 (37.5) | | 37 (40.7) |  | |
| Superior and inferior parts | 0 (0.0) | | 2 (2.2) |  | |
| None | 2 (12.5) | | 13 (14.3) |  | |
| Invasiveness |  | |  | 0.353 | |
| Yes | 5 (31.2) | | 16 (17.6) |  | |
| No | 11 (68.8) | | 75 (82.4) |  | |
| Extent of tumor resection |  | |  | 0.266 | |
| total resection | 15 (93.8) | | 72 (79.1) |  | |
| subtotal resection | 1 (6.2) | | 6 (6.6) |  | |
| partial resection | 0 (0.0) | | 13 (14.3) |  | |
| Diaphragma sellae sinking depth, mm | 7.13 ±6.00 | | 2.20±3.10 | < 0.001 | |
| Pituitary stalk deviation angle difference, (°) | 22.19±15.93 | | 7.54 ±12.21 | < 0.001 | |
| Length of the “measurable pituitary stalk”  （Preoperative） | 3.07±3.47 | | 4.34 ±3.69 | 0.203 | |
| length of the “measurable pituitary stalk”  （Postoperative） | 8.66±2.13 | | 5.69 ±3.25 | 0.001 | |
| Preoperative shape of pituitary stalk |  | |  | 0.523 | |
| curved | 7 (43.8) | | 51 (56.0) |  | |
| straight | 9 (56.2) | | 40 (44.0) |  | |
| Postoperative shape of pituitary stalk |  | |  | 0.337 | |
| curved | 2 (12.5) | | 25 (27.5) |  | |
| straight | 14 (87.5) | | 66 (72.5) |  | |
| **Laboratory examination variables** |  | |  |  | |
| Pathological tumor type |  | |  | 0.681 | |
| Gonadotropinoma | 7 (43.8) | | 33 (36.3) |  | |
| Null-cell adenoma | 4 (25.0) | | 13 (14.3) |  | |
| PRL cell adenoma | 0 (0.0) | | 12 (13.2) |  | |
| GH cell adenoma | 3 (18.8) | | 14 (15.4) |  | |
| ACTH cell adenoma | 1 (6.2) | | 8 (8.8) |  | |
| Plurihormona | 1 (6.2) | | 10 (11.0) |  | |
| TSH cell adenoma | 0 (0.0) | | 1 (1.1) |  | |
| Preoperative FT3, pmol/L | 3.62 ±0.86 | | 4.39 ±0.94 | 0.003 | |
| PostoperativeFT3, pmol/L | 2.80 ±0.79 | | 3.23 ±0.70 | 0.031 | |
| Preoperative FT4, pmol/L | 12.31 ±3.56 | | 13.53±3.38 | 0.188 | |
| Postoperative FT4, pmol/L | 13.29±2.64 | | 14.34 ±4.97 | 0.412 | |
| Preoperative TSH, mIU/L | 1.90 ±1.67 | | 1.76±1.37 | 0.719 | |
| Postoperative TSH, mIU/L | 0.35 ±0.24 | | 0.87 ±0.83 | 0.014 | |
| Preoperative cortisol, nmol/L | 8.77 ±7.34 | | 16.05 ±12.45 | 0.026 | |
| Postoperative cortisol, nmol/L | 8.77 ±7.34 | | 29.90 ±15.08 | 0.083 | |
| Preoperative ACTH, pg/ml | 17.56±10.93 | | 35.04±39.87 | 0.086 | |
| Postoperative ACTH, pg/ml | 23.43±28.91 | | 33.27 ±29.47 | 0.219 | |
| Preoperative serum sodium level, mEq/L | 135.94±8.77 | | 140.77 ±2.78 | < 0.001 | |
| Serum sodium on the first day after surgery, mEq/L | 138.38±4.59 | | 139.40 ±3.03 | 0.255 | |
| Serum sodium on the second day after surgery, mEq/L | 137.10 ±4.67 | | 140.64±3.28 | < 0.001 | |
| The difference in serum sodium levels before and 1st day after surgery, mEq/L | 1.37±3.26 | | -2.44 ±7.41 | 0.001 | |
| The difference inserum sodium levels before and 2nd day after surgery, mEq/L | 0.08±3.98 | | -1.16±8.05 | 0.34 | |
| Data are presented as number of patients (%). PRL, Prolactin; GH, growth hormone; ACTH, adrenocorticotropic hormone; TSH, thyroid-stimulating hormone; FT3, Free triiodothyronine; FT4, free thyroxine; PPBS, posterior pituitary bright spot; DI, diabetes insipidus. | | | | | |
